# Supplementary material for: Ferroptosis Inhibition Combats Metabolic Derangements and Improves Cardiac Function in Pulmonary Artery Banded Pigs
Source: bioRxiv. 2024 Oct 24:2024.04.24.590907. Preprint. [Version 2] doi: 10.1101/2024.04.24.590907 (PMC11526868; doi:10.1101/2024.04.24.590907)
Supplement: Supplement 1 [file media-1.docx]

**Supplemental Methods**

*Animal Groups and Treatment*: 8-10 week old Yorkshire Cross castrated male pigs were randomized into two treatment groups: ferrostatin-1 (PAB-Fer-1) or Placebo (PAB-Placebo). To improve translatability, vehicle (2% dimethyl sulfoxide, 50% polyethylene glycol, 5% Tween 80, and 43% double distilled water) or ferrostatin-1 (0.2 mg/kg, daily intramuscular injection) treatment started three weeks after PAB and continued for a total of three weeks. Five control pigs were purchased from vendor and housed at the University of Minnesota without any interventions. Animals studies were approved by the University of Minnesota Institutional Animal Care and Use Committee protocol number 2112-39666A. One control animal suffered a pericardial effusion during hemodynamic study so no cardiac MRI was performed. Two PAB-Vehicle animals were excluded: one due to infection and poor weight gain during the experimental timeline and one due to insufficient PAB severity. One PAB-Fer-1 animal died before end-point analysis and autopsy revealed it suffered a strangulated hernia, which was deemed to be the cause of death.

*Pulmonary Artery Banding Procedure*: Animals were initially sedated with Telazol (4 mg/kg) and Xylazine (0.8mg/kg) via intramuscular injection. After allowing the drugs to take effect, an ear catheter was placed for intravenous access. The IV catheter was then connected to an IV infusion of 0.9% Normal saline (NaCl). Anesthesia was induced by administering 1-2 mg/kg propofol IV to effect. Following evaluation for an appropriate depth of anesthesia, the animals were intubated with an appropriately sized endotracheal tube. Ceftiofur (5 mg/kg, IM) was administered once during induction for prophylactic antibiotic coverage.

When confirmed the animal reached a deep plane of anesthesia, 1.1 mg/kg succinylcholine chloride was administered IV. A left thoracotomy was performed through the 4^th^ intercostal space. The pericardium was opened parallel and anterior to the phrenic nerve and cradled with 2-0 braided polyester free ties. The main pulmonary artery was isolated proximal to the bifurcation. An umbilical tape was double looped around the pulmonary artery using a right angle. The umbilical tape was tightened, creating an hour glass shape, while the vital signs were monitored. When the animal appeared to be stable, a 19 gauge needle on a fluid filled pressure monitoring line was placed in the RV. The post-band RV pressure was recorded. Allowing up to 15 minutes ensuring the animal remained stable, the umbilical tape was tied and a clip was placed to secure the knot. The pericardium was loosely approximated, and the chest cavity was washed with a warm antibiotic solution. A chest tube was then placed in the chest through an intercostal space caudal to the thoracotomy, exteriorized through the skin and connected to a water sealed vacuum drainage reservoir. The rib, muscle, and skin layers were closed in standard fashion.

The animas were observed during the immediate post-operative period for bleeding. The animal were weaned from the ventilator to initiate spontaneous breathing. Supplemental oxygen was provided through the endotracheal tube. When the animal was breathing normally, substantial fluid was no longer draining from the chest tube, and negative thoracic pressure was established, the chest tube was removed. Once the animal was determined to be stable, it was moved to RAR post-operative care area. The endotracheal tube was removed when appropriate. Post-operative antibiotic (Clavamox 14 mg/kg BID for seven days) following the main pulmonary artery banding procedure was administered.

*Hemodynamic study*: The animals were initially sedated with Telazol (4 mg/kg) and Xylazine (0.8mg/kg) via IM delivery. After allowing the drugs to take effect, an ear catheter was placed for intravenous access. The IV catheter was then connected to an IV drip with 0.9% Normal saline (NaCl). Anesthesia was induced by administering 1-2 mg/kg propofol IV. Following evaluation for an appropriate depth of anesthesia, the animals were intubated with an appropriately sized endotracheal tube. The animals were placed on a table in the right lateral decubitus position. Mechanical ventilation was initiated at 10-15 breaths per minute at a tidal volume of 1.5 x body weight (kg), oxygen at 3 L/min, and isoflurane set between 1 and 3%, as needed, to maintain anesthesia. Vital signs were monitored (ECG, SpO2, EtCO2, temperature). When confirmed the animal reached a deep plane of anesthesia, arterial and venous interventional access was gained through the jugular vein and femoral artery. The artery and vein were accessed via a surgical cut down. Heparin (250 mg/kg, IV) was administered prior to introducing a vascular sheath. An appropriately sized introducer sheath was placed in the jugular vein and femoral artery lumen and secured. A pressure line was connected to the femoral artery introducer for systemic pressure monitoring. A 7F Swan-Ganz catheter was placed in the jugular vein introducer port to obtain hemodynamic data. The systemic artery pressure and right sided pressures were connected pressure transducers, which were displayed on AD instruments LabChart.

*Cardiac magnetic resonance imaging/angiography (MRI/MRA) examination*: All studies were performed after animals had hemodynamic study and thus all animals were intubated and sedated as described above. Studies were performed at the University of Minnesota using a Siemens 1.5 Tesla AERA scanner (Siemens, Malvern, PA) with phased-array coil systems. The examination included localizers to assess cardiac position and a standard segmented steady-state free-precession cine sequence to assess cardiac volumes and function. The imaging parameters were as followed: typical repetition time of 3.0–3.5 milliseconds, echo time of 1.2–1.5 milliseconds, in-plane spatial resolution of 1.8×1.4 millimeters, and temporal resolution of 35–40 milliseconds. Short-axis images were acquired with a slice thickness of 6 millimeters from the roof of the right atrium to the apex of the left ventricle. Long axis cines were obtained in the four-chamber, three-chamber and two-chamber views with dedicated two-chamber RV view. The cardiac magnetic resonance examination sequences were gated with electrocardiogram. Following this, contrast-enhanced magnetic resonance angiography was performed with contrast bolus timed to trigger at main pulmonary artery to evaluate the pulmonary artery banding severity. CMR analyses were performed using standard software (Precession by Heart Imaging Technologies, Durham, NC). Left and right atrial and ventricular end-diastolic and end-systolic volumes, ejection fractions, and mass were quantified by planimetry of the end-diastolic and end-systolic endocardial and end-diastolic epicardial borders on the short-axis cine images. All CMRI analyses were performed blindly by FK.

*Confocal Microscopy*: Confocal microscopy was performed on RV free wall sections from all animals included in the final analysis to evaluate RV cardiomyocyte hypertrophy. Briefly, RV free wall slides were de-paraffinized through xylene and ethanol washes, heated in Reveal Decloaker Buffer (BioCare Medical, Pacheco, CA) for 30 minutes. Sections were washed/blocked with 5% goat serum in PBS and then and stained WGA-488 at a dilution of 1:50 for 30 minutes at 37^o^C. Samples were washed with PBS, exposed to Hoechst stain, and treated with an autofluorescence quenching kit (Vector Laboratories). Finally, samples were mounted in Prolong Glass Antifade Mountant (Thermo Scientific). Images were collected on a Zeiss LSM 900 Airyscan 2.0 microscope and blindly analyzed by RM. RV cardiomyocyte diameter was determined using FIJI (NIH).

*Mitochondrial Ultrastructural Analysis*: RV free wall tissue was placed into fixative (4% paraformaldehyde + 1% glutaraldehyde in 0.1M phosphate buffered, pH 7.2 (PB)). After fixation, tissue was washed with PB, stained with 1% osmium tetroxide, washed in H_2_O, stained in 2% uranyl acetate, washed in H2O, dehydrated through a graded series of ethanol and acetone and embedded in Embed 812 resin. Following a 24 hour polymerization at 60°C, 0.1 µM ultrathin sections were prepared and post-stained with lead citrate. Micrographs were acquired using a JEOL 1400 Plus transmission electron microscope (JEOL, Inc., Peabody, MA) at 80 kV equipped with a Gatan Orius camera (Gatan, Inc., Warrendale, PA) at the Mayo Clinic. RV mitochondrial morphology was analyzed by RM, and cristae morphology scoring was performed as described(1).

*Quantitative Mitochondrial Proteomics*: TMT-16plex quantitative proteomics, performed at the University of Minnesota Center for Metabolomics and Proteomics, of RV mitochondrial enrichments (Abcam) evaluated RV mitochondrial protein regulation in *n*=4 control, *n*=6 PAB-Vehicle, and *n*=6 PAB-Fer-1 specimens as we have previously described (2-4). Protein abundance was quantified using Proteome Discoverer Software version 3.0 and used for statistical analysis as described below.

*RV Metabolomics/Lipidomics Analysis*: Global metabolomics analysis of frozen right ventricular free wall specimens on *n*=5 Control, *n*=7 PAB-Placebo, and *n*=6 PAB-Ferrostatin-1 was performed at the University of Minnesota Center for Metabolomics and Proteomics using the Biocrates’ MxP® Quant 500 kit. Approximately 100 mg of RV free wall was placed in 2.0 mL Precelly standard tubes and homogenized 3 times for 30 seconds at 5,800 rpm. Samples were centrifuged at 10,000*g* for 5 minutes at 4°C and supernatant was collected. 10µL of the extract was loaded onto a well insert. Classes of metabolites were determined using 50µL of 1:1:1:0.16 water:EtOH:pyridine:phenyl isothiocyanate solution and incubated for an hour. ABSciex QTRAP 5500 triple-quadrupole (Farmington, MA, USA) mass spectrometer was used to perform metabolomic assays.

*Statistical Analysis*: The primary end-point of this study was right ventricular ejection fraction (RVEF). We estimated a sample size of *n*=10 animals to detect a significant difference in RVEF, however interim analysis revealed the primary end-point was met so the study was terminated. Statistical analyses were performed using Prism 10.0 (GraphPad Software). Normality of data was determined using the Shapiro-Wilk test. If data were normally distributed and there was equal variance as determined using the Brown-Forsythe test, One-way analysis of variance with Tukey’s multiple-comparisons test was performed. If there was unequal variance, Brown-Forsythe and Welch analysis of variance with Dunnett multiple-comparisons test was completed. If the data were not normally distributed, the Kruskal-Wallis test and Dunn’s multiple-comparisons test were used when comparing three groups. P-values of <0.05 were considered to indicate statistical significance. Hierarchical cluster analyses, partial least squares discriminate analysis, and random forest classification of proteomics and metabolomics/lipidomics data were performed using MetaboAnalyst software. The relative abundance of each protein in the proteomics experiments were determined using Proteome Discover Software as we have previously described(2). Kyoto Encyclopedia of Genes and Genomes (KEGG) pathway analysis of the top 250 proteins most important for distinguishing the three experiment groups as determined by Random Forest Classification was performed using ShinyGO 0.76.3 (<http://bioinformatics.sdstate.edu/go/>). All pathways enriched were defined by a false discovery rate of *p*<0.05.

*Data Availability:* All data will be provided upon reasonable request. The raw proteomics (10.6084/m9.figshare.24878304) and metabolomics/lipidomics (10.6084/m9.figshare.24943449) data are currently available on Figshare.com with the listed DOI.

Supplemental Figure 1: Ferrostatin-1 counteracts pathological changes in the right heart despite no changes in RV afterload. Cardiac MRI examination revealed ferrostatin-1 reduced right ventricular end-diastolic volume indexed to body mass (A), the ratio of right ventricular end-diastolic volume to left ventricular end-diastolic volume (B), and tricuspid regurgitation severity (C). (D) Representative confocal micrographs stained with wheat germ agglutinin (purple) and DAPI (blue) staining of RV cross sections and subsequent quantification of cardiomyocyte cross-section area (E). (F) Three-dimensional reconstruction (above) and two-dimensional views of pulmonary artery magnetic resonance angiogram at the level of the aortic valve. (G) Quantification of the percent of pulmonary artery stenosis in PAB-Placebo and PAB-Fer-1 animals. (H) Hemodynamic evaluation of RV afterload as defined by right ventricular systolic pressure relative to systemic arterial systolic pressure. Ferroptosis inhibition mitigated pathological right atrial remodeling and dysfunction as right atrial end-diastolic volume relative to body weight (I), right atrial ejection fraction (J), and estimated right atrial-right ventricular coupling (K) were all improved with ferrostatin-1.


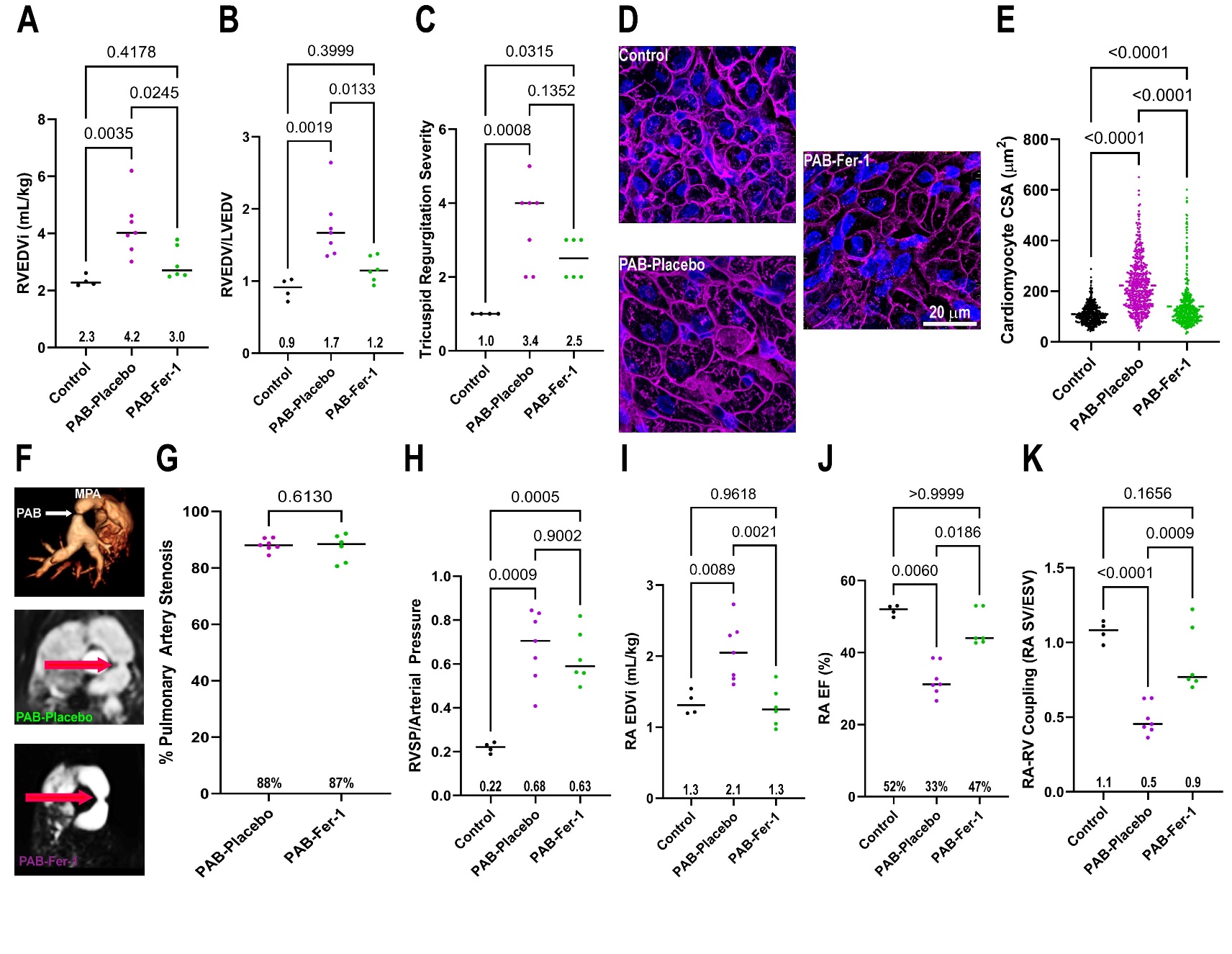


Supplemental Table 1: Results of comprehensive cardiac MRI examination

|  | Control (*n*=4) | PAB-Vehicle  (*n*=7) | PAB-Fer-1 (*n*=6) |
| --- | --- | --- | --- |
| RA EDV_i_ (mL/kg) | 1.3±0.2 | 2.1±0.4 | 1.3±0.3 |
| RA ESV_i_ (mL/kg) | 0.6±0.1 | 1.4±0.3 | 0.7±0.2 |
| RA SV_i_ (mL/kg) | 0.7±0.1 | 0.7±0.2 | 0.6±0.1 |
| RA EF (%) | 52±2 | 33±4 | 47±5 |
| RA SV/ESV | 1.1±0.1 | 0.5±0.1 | 0.9±0.2 |
| RV EDV_i_ (mL/kg) | 2.3±0.2 | 4.2±1.0 | 2.9±0.6 |
| RV ESV_i_ (mL/kg) | 1.1±0.2 | 3.0±1.1 | 1.8±0.5 |
| RV SV_i_ (mL/kg) | 1.4±0.1 | 1.2±0.2 | 1.2±0.2 |
| RV EF (%) | 59±2 | 30±7 | 41±5 |
| LV EDV_i_ (mL/kg) | 2.7±0.4 | 2.4±0.2 | 2.6±0.4 |
| LV ESV_i_ (mL/kg) | 1.1±0.3 | 1.3±0.2 | 1.2±0.2 |
| LV SV_i_ (mL/kg) | 1.5±0.2 | 1.2±0.1 | 1.3±0.2 |
| LV EF (%) | 53±3 | 48±5 | 52±3 |
| RVEDV/LVEDV | 0.9±0.2 | 1.7±0.4 | 1.2±0.2 |
| RV SV/ESV | 1.3±0.2 | 0.4±0.1 | 0.7±0.1 |
| TR Severity | 1±0 | 3.4±1.1 | 2.5±0.5 |
| Proximal PA CSA | NA | 2.3±0.3 | 2.5±0.5 |
| PA-Band CSA | NA | 0.3±0.03 | 0.3±0.1 |
| % Stenosis | NA | 88±2% | 87±5% |
| LGE Present at IVS | 0/4 | 6/7 | 3/6 |

Supplemental References

1. Lam J, Katti P, Biete M, Mungai M, AshShareef S, Neikirk K, et al. Cells: MDPI.

2. Kazmirczak F, Hartweck LM, Vogel NT, Mendelson JB, Park AK, Raveendran RM, et al. Intermittent Fasting Activates AMP-Kinase to Restructure Right Ventricular Lipid Metabolism and Microtubules. *JACC Basic Transl Sci.* 2023;8(3):239-54.

3. Mendelson JB, Sternbach JD, Doyle MJ, Mills L, Hartweck LM, Tollison W, et al. Multi-omic and multispecies analysis of right ventricular dysfunction. *J Heart Lung Transplant.* 2023.

4. Prisco SZ, Eklund M, Raveendran R, Thenappan T, and Prins KW. With No Lysine Kinase 1 Promotes Metabolic Derangements and RV Dysfunction in Pulmonary Arterial Hypertension. *JACC Basic Transl Sci.* 2021;6(11):834-50.
