# Supplementary material for: Ferroptosis Inhibition Combats Metabolic Derangements and Improves Cardiac Function in Pulmonary Artery Banded Pigs
Source: bioRxiv. 2024 Oct 24:2024.04.24.590907. Preprint. [Version 2] doi: 10.1101/2024.04.24.590907 (PMC11526868; doi:10.1101/2024.04.24.590907)

bioRxiv preprint doi: https://doi.org/10.1101/2024.04.24.590907; this version posted October 24, 2024. The copyright holder for this preprint (which was not certified by peer review) is the author/funder, who has granted bioRxiv a license to display the preprint in perpetuity. It is made available under aCC-BY 4.0 International license.

Supplemental Figure 1: Ferrostatin-1 partially mitigated RV insertion site late gadolinium enhancement. (A) Representative cMRI images of LGE. Arrows indicate RV insertion site LGE. (B) Proportion of animals with RV LGE.

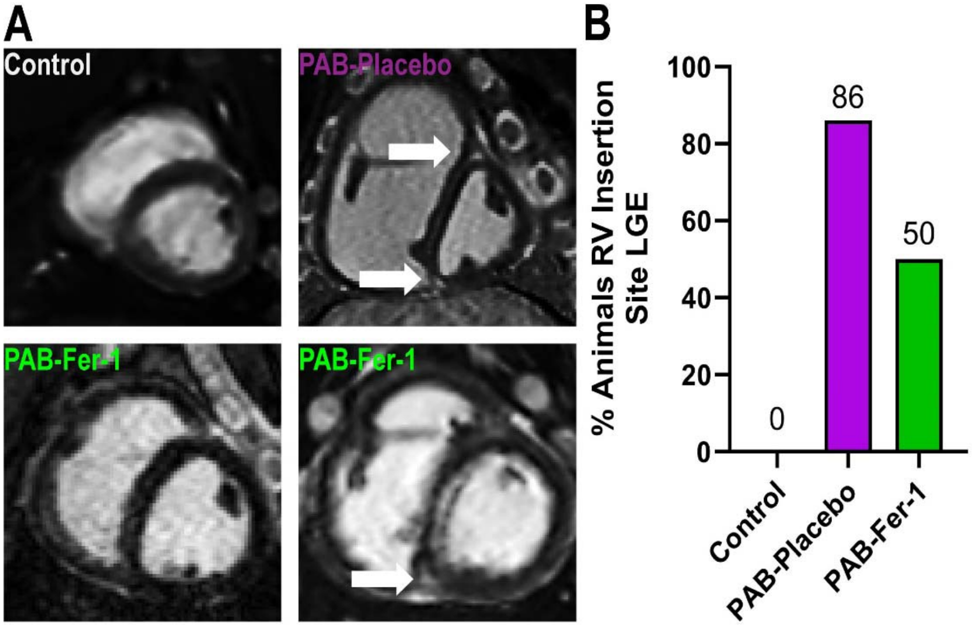

bioRxiv preprint doi: https://doi.org/10.1101/2024.04.24.590907; this version posted October 24, 2024. The copyright holder for this preprint (which was not certified by peer review) is the author/funder, who has granted bioRxiv a license to display the preprint in perpetuity. It is made available under aCC-BY 4.0 International license.

Supplemental Figure 2: Ferrostatin-1 Improved Mitochondrial Cristae Morphology. Representative electron micrographs of RV cardiomyocytes depicting mitochondrial cristae structure. Blinded quantification of mitochondrial cristae morphology. Ferrostatin-1 improved cristae morphology.

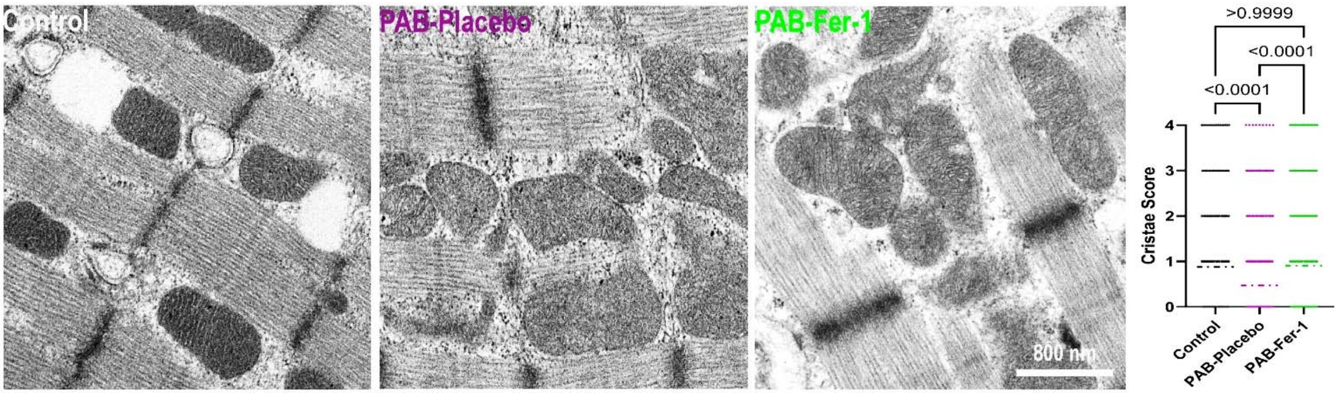

bioRxiv preprint doi: https://doi.org/10.1101/2024.04.24.590907; this version posted October 24, 2024. The copyright holder for this preprint (which was not certified by peer review) is the author/funder, who has granted bioRxiv a license to display the preprint in perpetuity. It is made available under aCC-BY 4.0 International license.

Supplemental Figure 3: Hierarchical cluster analysis of ferroptosis proteins.

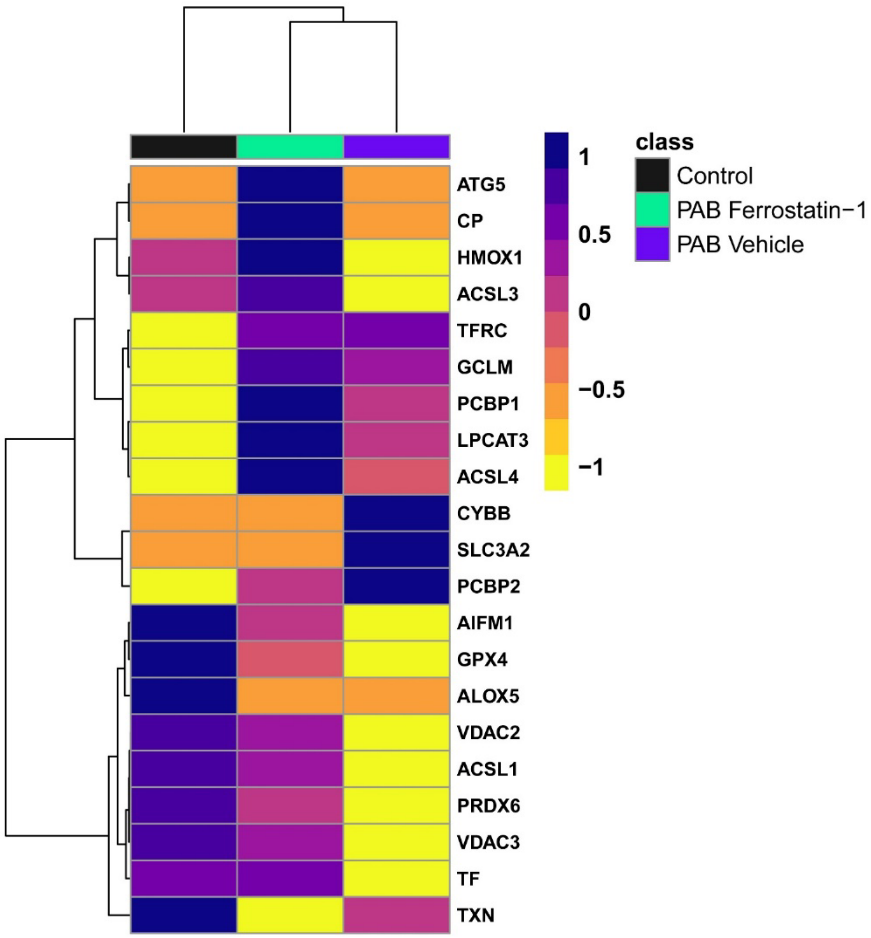

bioRxiv preprint doi: https://doi.org/10.1101/2024.04.24.590907; this version posted October 24, 2024. The copyright holder for this preprint (which was not certified by peer review) is the author/funder, who has granted bioRxiv a license to display the preprint in perpetuity. It is made available under aCC-BY 4.0 International license.

Supplemental Figure 4. Representative confocal micrographs stained with wheat germ agglutinin (purple) and DAPI (blue) staining of RV cross sections and subsequent quantification of cardiomyocyte cross-section area

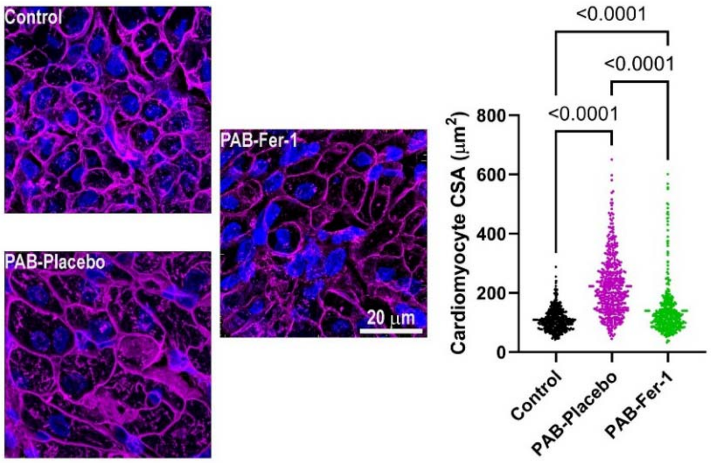

Supplement: Supplement 2 [file NIHPP2024.04.24.590907v2-supplement-2.pdf]
